# Metabolic and Nutritional Characteristics in Middle-Aged and Elderly Sarcopenia Patients with Type 2 Diabetes

**DOI:** 10.1155/2020/6973469

**Published:** 2020-11-04

**Authors:** Qinghua He, Xiuzhi Wang, Caizhe Yang, Xiaoming Zhuang, Yanfen Yue, Hongjiang Jing, Jing Hu, Mingxiao Sun, Lixin Guo

**Affiliations:** ^1^Department of Endocrinology, Beijing Hospital, National Center of Gerontology, Institute of Geriatric Medicine, Chinese Academy of Medical Sciences, 100730, China; ^2^Department of Endocrinology, Pinggu Hospital, Beijing Traditional Chinese Medicine Hospital, Beijing 10120, China; ^3^Department of Endocrinology, Air Force Medical Center, PLA, Beijing 100142, China; ^4^Department of Endocrinology, Fuxing Hospital Affiliated to Capital Medical University, Beijing 100038, China; ^5^Department of Nutriology, Pinggu Hospital, Beijing Traditional Chinese Medicine Hospital, Beijing 10120, China; ^6^Department of Nutriology, Air Force Medical Center, PLA, Beijing 100142, China; ^7^Department of Nutriology, Fuxing Hospital Affiliated to Capital Medical University, Beijing 100038, China; ^8^Department of Endocrinology, Beijing Eden Hospital, Beijing 100195, China

## Abstract

Sarcopenia is considered to be a new complication of type 2 diabetes (T2DM) leading to increased risk of adverse outcome. We performed a survey to evaluate glucose metabolism and nutritional status in sarcopenia patients with T2DM. Diabetic participants aged ≥50 years were grouped into a probable sarcopenia group with low muscle strength (*n* = 405) and a nonsarcopenia group with normal muscle strength (*n* = 720) according to the revised recommendations from EWGSOP2 (2018). Compared to the controls, the probable sarcopenia participants were older and had lower waist-to-hip ratio and BMI, longer diabetes duration, higher fasting plasma glucose level and glycosylated hemoglobin (HbA1c), decreased estimated glomerular filtration rate and lower bone mineral content, lower fatless upper arm circumference, lower appendicular skeletal muscle mass index (ASMI), and muscle quality in both genders. Multivariable logistic regression analysis showed increased age, male, low BMI, and increased HbA1c, combined with diabetic nephropathy and decreased serum albumin levels, were risk factors associated with low muscle strength in diabetes patients. In conclusion, diabetic patients with sarcopenia had worse glucose metabolism and nutritional status, decreased renal function and reduced muscle quality ,and muscle mass with a greater likelihood of osteoporosis, who need an overall health management to improve outcomes. This clinical trial registration is registered with the Chinese Clinical Trial Registry, ChiCTR-EOC-15006901.

## 1. Introduction

Sarcopenia is a progressive and generalized skeletal muscle disorder that is associated with an increased likelihood of adverse outcomes, including falls, fractures, physical disability, and mortality [1] and is preventable and treatable in the early stage [2]. It was originally defined as a decrease in muscle mass related to aging. However, studies in recent decades [3–5] showed that decreased muscle strength was more important than reduced muscle mass in predicting morbidity and mortality, which led to updates to the definition of sarcopenia and diagnostic strategies by the European Working Group on Sarcopenia in Older People (EWGSOP2) in 2018 [6]. Sarcopenia is now considered a skeletal muscle disease, with low muscle strength overtaking the role of low muscle mass as a principal determinant [3, 7]. Specifically, probable sarcopenia is considered when low muscle strength is detected, and confirmed sarcopenia is diagnosed by the presence of low muscle quantity or quality. Sarcopenia is always considered to be multifactorial and associated with multiple chronic diseases. Type 2 diabetes (T2DM), an increasingly prevalent metabolic disease worldwide, has been reported to result in a more rapid decline in muscle mass, strength, and functional capacity [8]. T2DM was thought to be an important predictive factor of sarcopenia [9], and the presence of sarcopenia with T2DM presents an increased risk of physical disability, changes in mental health, frailty, and increasing dependency [10]. However, to date, few studies have investigated the metabolic characteristics of sarcopenia patients with T2DM. The aim of our study was to evaluate the characteristics of glucose metabolism and nutritional status in middle-aged and elderly sarcopenia patients with T2DM. We analyzed the data collected from a multicenter, cross-sectional survey study designed to estimate the prevalence of sarcopenia in T2DM adults. In the present study, a large group of adult patients with T2DM were divided into a probable sarcopenia group with low muscle strength and a nonsarcopenia group with normal muscle strength. Anthropometric measurements, body composition, glucose metabolism, laboratory indicators, and nutritional status were compared between the two groups.

## 2. Research Design and Methods

### 2.1. Study Design and Participants

Data were collected from a multicenter, cross-sectional survey study designed to estimate the prevalence of sarcopenia in adults with type 2 diabetes recruited from the endocrinology departments of nine different hospitals in Beijing, China, from January 2016 to March 2018. The nine research centers consisted of five urban hospitals and four suburban hospitals selected by a systematic random sampling method. The inclusion criteria were as follows: patients aged 50 years or older with a previous diagnosis of type 2 diabetes. According to the World Health Organization definition, previously diagnosed type 2 diabetes was defined as having either fasting plasma glucose (FPG) ≥ 7.0 mmol/l or 2-h PG ≥11.1 mmol/l. The exclusion criteria included the following: (1) serious systemic diseases including severe hepatic insufficiency, moderate to severe renal insufficiency, or cardiac insufficiency; (2) tuberculosis; (3) severe depression, schizophrenia, or other mental illness; (4) cognitive disability or an inability to record in the diet diary and cooperate with the examination; (5) implantation with metal stent or pacemaker in vivo, which would affect the accuracy of body composition analysis; and (6) current or recent weight loss surgery. The study was approved by the Beijing Hospital Ethics Committee (Approval No. 2015BJYYEC-052-02), and informed consent was obtained from all participants. A total of 1125 participants who met the aforementioned criteria were included in the current data analysis. According to the recommendation of the revised European consensus on definition and diagnosis [6], low muscle strength was identified as probable sarcopenia. All populations were divided into two groups: a probable sarcopenia group with low muscle strength and a nonsarcopenia control group with normal muscle strength. We compared the differences in metabolic characteristics and anthropometric measurements between the two groups stratified by sex.

### 2.2. Questionnaire Survey

A standardized questionnaire was designed to collect patient information, including demographic data such as birth date, sex, habits and customs, alcohol drinking status (current drinker or not), smoking status (current smoker or not), the time of onset of diabetes mellitus, and medication for diabetes, including oral hypoglycemic drugs or insulin. In addition, some chronic complications, including cardiovascular disease, hypertension, diabetic peripheral vascular disease, diabetic peripheral neuropathy, and diabetic nephropathy, were self-reported or recorded from medical records. A team consisting of endocrinologists, nutritionists, educational nurses, and check-up physicians was involved in the study at each research center. The endocrinologist was responsible for data collection, and the nutritionist and educational nurses were in charge of dietary records and analysis. The check-up physician was responsible for anthropometric and body composition measurements. A standardized study protocol manual was distributed to every researcher, and all investigators had to receive unified training before the study to reduce bias among researchers.

### 2.3. Anthropometric and Body Composition Measurements

Body weight and height were measured using a digital floor scale and a wall-mounted stadiometer to the nearest 0.1 cm and 0.1 kg, respectively, with the patient wearing light clothes and without shoes. Body mass index (BMI) was calculated as weight in kilograms divided by the square of height in meters (kg/m^2^). Body composition was determined by a bioimpedance analyzer using an Inbody 720 (Biospace, Korea). We recorded trunk muscle mass, appendicular and whole body skeletal muscle mass, fat mass, and percentage of body fat. The appendicular skeletal muscle mass index (ASMI) was calculated by dividing the appendicular skeletal muscle mass by the height squared (kg/m^2^). The handgrip strength of each hand was measured 3 times using a digital grip strength dynamometer (EH101, Zhejiang Province, China). Trained medical technicians instructed the participants who were sitting to hold the dynamometer with the distal interphalangeal finger joints of the hand at 90° to the handle and to squeeze the handle as firmly as they could. After the participants stood up slowly, the handgrip strength was measured during expiration. Study participants performed 3 attempts per hand, with a 1 minute rest period between each attempt to reduce the effect of fatigue due to repetition. The measurements of the handgrip strength were presented as an average of the 3 measurements with either hand [11].

We categorized the participants according to weight based on the World Health Organization criteria [12]: normal weight (BMI < 25 kg/m^2^), overweight (25 kg/m^2^ BMI < 30 kg/m^2^), and obesity (BMI ≥ 30 kg/m^2^). Muscle quality [13] was measured by ratios of muscle strength to the appendicular skeletal muscle mass.

### 2.4. The Cutoff Point for Diagnosis of Sarcopenia

The Asian-specific cutoff point for diagnosis of low muscle mass and low muscle strength was according to the recommendation of the Asian Working Group for Sarcopenia (AWGS) in 2014 [14]. Low muscle mass was determined as the ASMI below the lower quintile of the homogeneity, same sex, and healthy young reference group. The cutoff points of ASMI were based on the results of healthy young staff members at Beijing Hospital in China, who received routine health check-ups and body composition examinations by a bioimpedance analyzer. This cohort included 402 volunteers whose vocations were doctor and nurse (102 males and 300 females), aged between 18 and 35 years old, with BMI values between 18.5 kg/m^2^ and 24.0 kg/m^2^. Their ASMIs were calculated, and the lower quintile of ASMI was 7.18 kg/m^2^ and 5.73 kg/m^2^ in men and women, respectively. Participants with an ASMI less than 7.18 kg/m^2^ in men or 5.73 kg/m^2^ in women were considered to have low muscle mass. Low muscle strength was defined as the handgrip strength below the lower quintile of the same sex subjects in this study, and the lower quintile cutoff value for muscle strength was 29.5 kg for males and 21.2 kg for females.

### 2.5. Laboratory Measurements

The study participants were instructed to maintain an overnight fast of at least 10 hours before blood samples were collected. Routine blood examinations, including red blood cells, hemoglobin, and hematocrit, were performed using a hematology analyzer (SYSMEX XN-20 AI, USA). Serum albumin, fasting plasma glucose level, and serum creatinine were measured by an automatic biochemical analyzer (Beckman Coulter AU5400, USA). Fasting plasma glucose was determined by the glucose oxidase method. Glycated hemoglobin A1c (HbA1c) was measured by high-performance liquid chromatography (HPLC) (Premier HB9210, Trinity Biotech, Kansas, USA). The glomerular filtration rate was estimated according to the Cockcroft-Gault equation: eGFR = [(140 − age) × body weight (kg)]/[0.818 × creatinine (*μ*mol/l)] (∗0.85 in female).

### 2.6. Dietary Records and Analysis

All participants had to record a diet diary for three continuous days, including two working days and one weekend day, in accordance with the guidance of a nutritionist and educational nurses. The weight of each type of food that is eaten during the three days, including staple foods, vegetables, meats, and snacks, should be recorded. We calculated the total energy, carbohydrate, protein, and fat intake per day and the proportion of calories supplied by protein, carbohydrate, and fat with nourishment analysis software (V4.0.3, Zhending Health Technology Co, Shanghai, China). The total energy was adjusted by body weight, which was calculated by the total energy daily (kcal) divided by the ideal body weight (kilogram). The ideal body weight was calculated by 105 subtracted from height (centimeter).

### 2.7. Statistical Analysis

Statistical analysis was performed using SPSS for Windows, version 18.0 (SPSS, Chicago, IL). All parameters were tested for a normal distribution. Normally distributed continuous data are presented as the means ± standard deviation, and categorical variables are presented as numbers and percentages (%). An independent sample *T* test was used to compare the means of two groups, and a chi-square test was used to compare the percentage. Data with a skewed distribution were presented as the median (25th-75th percentile) and tested with the Kruskal-Wallis test. A multivariable logistic regression analysis using the backward stepwise likelihood ratio method to estimate the risk factors for low muscle strength was fitted with candidate variables including age, sex, BMI, diabetes duration, glycosylated hemoglobin, treatment regimen, nutrient intake per day, chronic complications of diabetes mellitus, current drinking, and current smoking. The statistical tests were 2-tailed, and a *P* value of <0.05 was considered statistically significant.

## 3. Results

### 3.1. The Baseline Characteristics of the Two Groups

Table 1 shows the baseline characteristics and chronic complications in the two groups. The prevalence of low muscle strength was 36.0% in all diabetic participants, with 50.3% in men and 20.4% in women. Of the 405 patients with probable sarcopenia, there were 295 men and 110 women, and of the 720 patients in the control group, there were 291 men and 429 women. Compared to the nonsarcopenia controls, male and female probable sarcopenia patients were older and had lower waist-to-hip ratios and BMIs. There were more male patients with complications of diabetic peripheral neuropathy and diabetic nephropathy, more male participants treated with insulin, and fewer male participants treated with oral hypoglycemic agents in the probable sarcopenia group than in the control group; however, such differences were not observed in the female subgroup.

### 3.2. Laboratory Measurement Analysis

Table 2 shows the laboratory measurements in the two groups stratified by sex. The fasting plasma glucose level and glycosylated hemoglobin (HbA1c) level were significantly higher in the probable sarcopenia group than in the control group for both men and women. In the probable sarcopenia group, the red blood cell count, hemoglobin level, and hematocrit and serum albumin were obviously lower in men, while the serum creatinine level was higher in women than in the control group. The estimated glomerular filtration rate (eGFR) declined significantly for both men and women in the probable sarcopenia group compared with those in the control group.

### 3.3. Dietary Intake Analysis in Participants with Type 2 Diabetes Mellitus

Table 3 shows intake of three major nutrients per day in the probable sarcopenia patients and nonsarcopenia controls stratified by sex. In all participants with type 2 diabetes, the average total calorie intake per day was 29.8 ± 7.4 kcal/kg/d, the dietary protein intake was 1.13 ± 0.34 g/kg/d, and the calorie percentage supplied by carbohydrate, protein, and fat was 53.8 ± 7.7%, 15.6 ± 3.9%, and 30.8 ± 6.9%, respectively. The calorie percentage supplied by carbohydrate was lower [(54.4 ± 8.5)% vs (53.0 ± 7.4)%, *P* = 0.03], while the percentage supplied by fat [(29.7 ± 7.6)% vs (31.4 ± 6.9)%, *P* = 0.005] was higher in the probable sarcopenia group than in the control group, and the difference was significant in men but not in women. There was no difference in total calorie and dietary protein intake adjusted by the body weight between the probable sarcopenia group and the control group.

### 3.4. Body Composition Analysis between Two Groups

Table 4 shows the body composition analysis of the T2DM participants in the probable sarcopenia group and control group stratified by sex. Compared to the control group, the probable sarcopenia group had significantly lower bone mineral content, basal metabolic rate, fatless upper arm circumference, appendicular skeletal muscle mass, appendicular skeletal muscle mass index, and muscle quality in both the male and female subgroups, while there was no difference in the body fat percentage between the two groups. The prevalence of low muscle mass was obviously elevated in the probable sarcopenia group compared with the control group in both sexes.

### 3.5. The Risk Factors for Low Muscle Strength from Multivariable Logistic Regression Analysis

The Box-Tidwell method test for linearity suggested that the continuous independent variables were linearly associated with the logit of low muscle strength probability. After the variable selection procedure and adjustment for smoking and drinking status, the final logistic model to estimate the probability of low muscle strength included six variables that were statistically significant. In the multivariable logistic regression analysis, increased age, male sex, low BMI, increased glycosylated hemoglobin, diabetic nephropathy (OR = 1.439, 95% CI: 1.033~2.006, *P* = 0.031), and decreased serum albumin levels (OR = 0.917, 95% CI: 0.883~0.953, *P* < 0.001) were risk factors associated with low muscle strength in type 2 diabetes patients.  Table 5 shows the six risk factors for probable sarcopenia in diabetes patients estimated by multivariable logistic regression analysis.

## 4. Discussion

The present study found that diabetic patients with sarcopenia were much older and had lower BMI and worse glucose metabolism than nonsarcopenia diabetic patients. In addition, diabetic patients with sarcopenia had a lower appendicular skeletal muscle mass (ASM) and ASMI with a higher prevalence of low muscle mass in both male and female patients. T2DM patients are known to be at high risk of developing sarcopenia due to metabolic abnormalities and insulin resistance [8, 15]. Not only the HbA1c level but also glucose fluctuations were significantly associated with a low muscle mass, low grip strength, and slow walking speed [16].

A metabolic consequence of uncontrolled hyperglycemia is catabolism, which is accompanied by muscle protein breakdown and inadequate energy use, potentially resulting in decreased muscle mass and poor muscle function [8]. Conversely, the skeletal muscle not only is responsible for the physical function but also is a metabolically active tissue [17]. The whole body skeletal muscle is the largest organ responsible for insulin-mediated glucose disposal in humans, and the progressive loss of the skeletal muscle might lead to diminished insulin-mediated glucose disposal and exacerbated insulin resistance independent of obesity in sarcopenia, resulting in severe glucose abnormalities [18]. T2DM has been implicated as both a cause and consequence of sarcopenia through altered muscle mass, increased localized inflammation, and arise through inter- and intramuscular adipose tissue accumulation [19]. Additionally, in our study, almost 48.2% of male and 55.5% of female sarcopenic diabetic patients were overweight or obese. Sarcopenic obesity [20] is a condition of reduced lean body mass in the context of excess adiposity. Obesity exacerbates sarcopenia, increases the infiltration of fat into muscle, and lowers physical function [21]. It is possible that alterations of muscle composition with increased fat infiltration into the skeletal muscle or muscle steatosis aggravate insulin resistance and then deteriorate glucose metabolism. As Srikanthan et al. [22] found, sarcopenia was associated with increased insulin resistance in both nonobese and obese individuals, which was at least partly attributed to poor glucose metabolism in sarcopenic patients in our study.

In addition to significant differences in skeletal muscle mass, we observed significant differences in muscle quality between the two groups. The muscle quality in the sarcopenia group decreased obviously compared with that in the age-matched nonsarcopenia control group in both men and women. As muscle strength is an important factor determining the level of functional capacity, alternatively, the term muscle quality has been applied to ratios of muscle strength to appendicular skeletal muscle mass [13] and has been consistently used to assess the muscle function with different body sizes, such as those with diabetes. Muscle quality is a more reasonable indicator of the contractile function of the skeletal muscle than crude muscle strength, which is largely dependent on the quantity of muscle mass. In Western populations, despite the larger muscle mass, muscle quality was consistently lower in older adults with type 2 diabetes, regardless of sex or muscle groups examined.

It is generally accepted that the musculoskeletal system is an integrated cosystem. Our results showed that the bone mineral content in the probable sarcopenia group decreased obviously compared with that in the nonsarcopenia group, both in men and women, implying that sarcopenic diabetic patients were more likely to suffer from osteoporosis. As is known, bone and muscle are closely interconnected with each other not only via their adjacent surfaces but also chemically and metabolically [23]. Clinically, the combination of osteoporosis and sarcopenia in diabetes patients is associated with significant physical disability and exacerbated negative health outcomes such as falls, fractures, loss of independence in later life, and increased mortality [24]. It was suggested that when sarcopenia was synchronic with osteoporosis, more prevention and treatment measures were urgently needed.

Malnutrition has been reported to be an important etiology that leads to sarcopenia in diabetic patients. In the current study, we found that probable sarcopenia patients had lower hemoglobin, hematocrit, and albumin levels in men, and the fatless circumference of the upper arm decreased significantly in both sexes compared with that in the control group, suggesting that sarcopenic diabetic patients were at higher risk of malnutrition. It could be partly speculated that the blunting of the muscle protein synthetic response to food intake is even more pronounced in an insulin-resistant state, resulting in a more rapid decline in skeletal muscle mass in sarcopenic diabetic patients. A low hemoglobin level is an independent risk factor for increased mortality and a lowered quality of life in the elderly. Decreased serum albumin and hemoglobin implied insufficient protein intake, which will increase the risk of malnutrition-related mortality. In addition, we found that most of the participants in our study had an unbalanced nutrition intake with excessive total calories, deficient dietary protein, and excessive dietary fat intake. An inadequate protein intake may offset the muscle protein synthetic response, which is vital for maintaining and regaining muscle mass in sarcopenia. Evidence suggests that nutrition therapy plays an integral role in the prevention and treatment of sarcopenia, but there is no ideal percentage of calories from carbohydrate, protein, and fat for all people; therefore, the macronutrient distribution in meal planning should be based on individuals. Most international clinical guidelines [25] recommended that the total calorie intake was 25-30 kcal/kg/day for normal weight diabetic patients and 20-25 kcal/kg/day for overweight and obese patients aimed to control body weight. The American ADA guideline [25] recommended that daily energy intake was 1200-1500 kcal for females and 1500-1800 kcal for males. To improve health in individuals, the recommended dietary protein intake was 1–1.5 g/kg/day or 15–20% of total calories for patients without diabetic kidney disease. For those with diabetic kidney disease (with albuminuria and/or reduced estimated glomerular filtration rate), dietary protein should be maintained at the recommended daily allowance of 0.8 g/kg body weight/day, and high-quality protein components such as whey protein and other animal proteins are more beneficial for preventing the prevalence of sarcopenia.

Another important finding of this study was that sarcopenic diabetic patients had a lower eGFR than the controls, suggesting decreased renal function in sarcopenic patients. The glomerular filtration rate (GFR) is an important index relative to the kidney function and plays a significant role in the detection, progression, and treatment of chronic kidney disease. A previous study [26] suggested that muscle mass might be a protective factor the kidney function. Participants with high muscle mass exhibited higher eGFR [27], and their ASMI was positively correlated with eGFR and negatively correlated with the urinary albumin/creatinine ratio (UACR) [28]. Moreover, their body fat mass and distribution induced a decline in eGFR, showing that it was a risk factor for eGFR and chronic kidney disease independent of skeletal muscle mass in healthy young men as well as nondiabetic and diabetic populations [29]. The pathophysiological mechanism of sarcopenia and kidney function damage was multifactorial. Their shared underlying mechanisms included insulin resistance [30], endothelial dysfunction [31, 32], inflammation, oxidative stress, and activation of the renin-angiotension system (RAS). As loss of skeletal muscle mass is persistent in diabetes patients, further screening should be required to assess renal dysfunction.

The present study had several limitations that must be addressed. First, because of the inherent limitations of the cross-sectional study design, we could not determine a causal relationship between sarcopenia and those correlated metabolic variables. Second, physical activity, which was an important factor associated with sarcopenia, was not analyzed in our study because of a lack of information in the questionnaire. Third, since age might influence many parameters as a confounded variable, which was significantly higher in the sarcopenia group, some of the key variables of the study should be analyze stratified by age. However, the sample size would become too small to compare when stratified by age and sex. In order to exclude confounding factors such as age, a multivariable logistic regression analysis was used to estimate the risk factors for low muscle strength. Last, the current results were carried out on a study population of Chinese adults with type 2 diabetes. Our findings should therefore be confirmed in other ethnic and nondiabetic populations in the future.

In conclusion, our study found that compared to nonsarcopenic diabetic controls, sarcopenic diabetic patients were older, had lower BMI, and worse nutritional status with an unbalanced nutrition intake and demonstrated deteriorated glucose metabolism, decreased renal function, and a reduction in muscle quality and skeletal muscle mass with a greater likelihood of osteoporosis. Diabetic patients with sarcopenia should receive more attention and need overall health management to improve their outcomes.

## Figures and Tables

**Table 1:** Characteristics of the T2DM participants in the sarcopenia group and control group stratified by sex.

	Men				Women			
	Control group	Sarcopenia group	*t*/*X*^2^/*Z*	*P*	Control group	Sarcopenia group	*t*/X^2^/*Z*	*P*
*N*	291	295			429	110		
Age (years)	60.3 ± 7.4	64.6 ± 9.3	-6.139	≤0.001	62.1 ± 7.2	64.9 ± 9.1	-3.017	0.003
BMI (kg/m^2^)	26.2 ± 3.4	25.3 ± 3.3	3.252	0.001	26.0 ± 3.2	25.6 ± 3.5	1.057	0.291
<25	119 (40.9%)	150(50.8%)			177 (41.3%)	49 (44.5%)		
25-30	135 (46.4%)	123(41.7%)			209 (48.7%)	49 (44.5%)		
≥30	37 (12.7%)	22(7.5%)			43 (10.0%)	12 (10.9%)		
Waist-to-hip ratio	0.93 ± 0.06	0.92 ± 0.07	1.963	0.050	0.93 ± 0.06	0.92 ± 0.06	1.956	0.051
Diabetes duration (years)	10.0 (5.1, 15.4)	10.6 (5.5, 17.8)	-1.850	0.064	10.2 (5.2, 15.2)	11.2 (6.0, 15.7)	-1.722	0.085
Current smoker, *n* (%)	108 (37.2%)	101 (34.2%)	0.575	0.448	12 (2.8%)	5 (4.5%)	0.876	0.349
Current drinker, *n* (%)	157 (54%)	125 (42.4%)	7.867	0.005	32 (7.5%)	4 (3.6%)	2.053	0.152
Hypertension, *n* (%)	173 (59.5%)	188 (63.7%)	1.134	0.287	236 (55.0%)	67 (60.9%)	1.237	0.266
Coronary heart disease, *n* (%)	49 (16.8%)	58 (19.7%)	0.782	0.377	65 (15.2%)	26 (23.6%)	4.492	0.034
Peripheral neuropathy, *n* (%)	107 (36.8%)	154 (52.2%)	14.126	≤0.001	178 (41.5%)	45 (40.9%)	0.012	0.912
Diabetic nephropathy, *n* (%)	93 (32.0%)	134 (45.4%)	11.192	0.001	133(31.0%)	34(30.9%)	≤0.001	0.985
Oral hypoglycemic agents, *n* (%)	221 (75.9%)	201 (68.1%)	4.433	0.035	323 (75.3%)	91 (82.9%)	2.718	0.099
Insulin treatment, *n* (%)	104 (35.7%)	151 (51.2%)	14.222	≤0.001	164 (38.2%)	40 (36.4%)	0.129	0.719

Notes: data were expressed as the mean ± standard deviation or % and median (25th–75th percentile).

**Table 2:** Laboratory measurements of T2DM participants in the sarcopenia group and control group stratified by sex.

	Men				Women			
	Control group	Sarcopenia group	*t*	*P*	Control group	Sarcopenia group	*t*	*P*
FPG (mmol/l)	7.6 ± 2.4	8.5 ± 2.7	-3.352	0.001	7.7 ± 2.7	8.8 ± 3.1	-2.660	0.008
HbA1c (%)	6.9 ± 1.5	7.8 ± 1.7	-6.552	≤0.001	7.4 ± 1.9	8.2 ± 2.0	-3.151	0.002
Red blood cells (10^12^/l)	4.8 ± 0.4	4.5 ± 0.5	6.207	≤0.001	4.4 ± 0.4	4.3 ± 0.4	0.700	0.484
Hemoglobin (g/l)	147.7 ± 13.8	138.7 ± 14.1	7.734	≤0.001	130.8 ± 12.7	130.7 ± 12.4	0.053	0.958
Hematocrit (%)	43 ± 4.7	40.3 ± 4.2	7.062	≤0.001	38.9 ± 3.7	38.9 ± 3.8	0.004	0.997
Albumin (g/l)	43.9 ± 4.2	41.0 ± 3.3	9.133	≤0.001	42.9 ± 3.9	42.9 ± 3.9	-0.104	0.917
Creatinine (*μ*mol/l)	71.4 ± 13	71.1 ± 18.6	0.222	0.824	55.6 ± 13.4	59 ± 16.2	-2.315	0.021
eGFR (ml/min)	109.6 ± 30.1	101.8 ± 31.1	3.092	0.002	101.7 ± 28.1	89.1 ± 29.1	4.162	≤0.001

Notes: data were expressed as the mean ± standard deviation or % and median (25th–75th percentile). Abbreviations: FPG: fasting plasma glucose; HbA1c: glycated hemoglobin; eGFR: estimated glomerular filtration rate; TG: triglycerides; TC: total cholesterol; HDL-C: high-density lipoprotein cholesterol; LDL-C: low-density lipoprotein cholesterol.

**Table 3:** Intake of three primary nutrients per day in T2DM participants in the sarcopenia group and control group stratified by sex.

	Men				Women			
	Control group	Sarcopenia group	*t*	*P*	Control group	Sarcopenia group	*t*	*P*
Total calories (kcal/d)	1916.6 ± 478.1	1965.1 ± 513.7	-1.185	0.237	1618.6 ± 361.2	1513.4 ± 348	2.745	0.006
Total calories adjusted by BW (kcal/kg/d)	29.1 ± 7.4	30.3 ± 8.1	-1.946	0.052	29.9 ± 7	29.5 ± 7.2	0.511	0.610
Protein intake adjusted by BW (g/kg/d)	1.15 ± 0.37	1.14 ± 0.34	0.449	0.654	1.12 ± 0.33	1.10 ± 0.34	0.666	0.505
Calories by carbohydrate (%)	54.4 ± 8.5	53.0 ± 7.4	2.176	0.030	54.1 ± 6.9	53.6 ± 8.8	0.563	0.574
Calories by protein (%)	16.1 ± 4.2	15.7 ± 3.4	1.209	0.227	15.0 ± 3.0	16.0 ± 6.7	-1.431	0.155
Calories by fat (%)	29.7 ± 7.6	31.4 ± 6.9	-2.823	0.005	31.1 ± 6.1	31.3 ± 7.2	-0.342	0.733

Notes: data were expressed as the mean ± standard deviation. Abbreviation: BW: body weight.

**Table 4:** Body composition in T2DM participants in the sarcopenia group and control group stratified by sex.

	Men				Women			
	Control group	Sarcopenia group	*t*/*X*^2^	*P*	Control group	Sarcopenia group	*t*/*X*^2^	*P*
Body fat (%)	27.0 ± 5.7	26.6 ± 6.6	0.819	0.413	35.2 ± 5.7	36.0 ± 6.1	-1.182	0.238
Bone mineral content (kg)	3.1 ± 0.4	3.0 ± 0.4	3.769	≤0.001	2.5 ± 0.3	2.3 ± 0.3	4.273	≤0.001
Basal metabolic rate (kcal/d)	1569.5 ± 133.1	1518.3 ± 153.1	4.322	≤0.001	1287.7 ± 112.4	1230.1 ± 102.3	4.876	≤0.001
Fatless circumference of upper arm (cm)	27.2 ± 1.9	26.3 ± 2.1	5.242	≤0.001	24.4 ± 1.7	23.7 ± 1.7	3.634	≤0.001
ASM (kg)	23.6 ± 2.9	22.5 ± 3.4	4.134	≤0.001	17.1 ± 2.6	15.8 ± 2.4	5.087	≤0.001
ASMI (kg/m^2^)	8.0 ± 0.7	7.7 ± 0.8	4.654	≤0.001	6.7 ± 0.7	6.4 ± 0.8	4.033	≤0.001
Muscle quality	12.0 ± 2.6	9.5 ± 2.1	12.972	≤0.001	12.4 ± 2.5	9.0 ± 1.7	16.775	≤0.001
Low muscle mass, *n* (%)	32 (11.0%)	75 (25.4%)	20.429	≤0.001	29 (6.8%)	21 (19.1%)	15.818	≤0.001

Notes: data were expressed as the mean ± standard deviation or %. Abbreviations: ASM: appendicular skeletal muscle mass; ASMI: appendicular skeletal muscle mass index.

**Table 5:** The risk factors for sarcopenia in diabetes, estimated using multivariable logistic regression analysis.

Variables	*β*	SE	Wald*χ*^2^	*P*	OR (95% CI)
Sex (compared to men)	-1.63	0.165	97.886	≤0.001	0.196 (0.142~0.271)
Age (years)					
50~59					1
60~74	0.417	0.152	7.531	0.006	1.517 (1.127~2.043)
≥75	0.938	0.254	13.624	≤0.001	2.555 (1.553~4.205)
BMI (kg/m^2^)					
<24					1
24~28	-0.185	0.165	1.261	0.261	0.831 (0.601~1.148)
≥28	-0.382	0.194	3.875	0.049	0.683 (0.467~0.998)
HbA1c (%)					
<7					1
7~10	0.084	0.244	0.12	0.729	1.088 (0.675~1.755)
≥10	0.334	0.161	4.292	0.038	1.396 (1.018~1.915)
Diabetic nephropathy	0.364	0.169	4.628	0.031	1.439 (1.033~2.006)
Albumin	-0.087	0.019	19.87	≤0.001	0.917 (0.883~0.953)

## Data Availability

The research data used to support the findings of this study are available from the corresponding author upon request. The corresponding author email: glx1218@163.com

## Additional Points

*Prior Presentation*. Parts of this study were presented in abstract form at the IDF Congress, Busan, South Korea, 2-6 December, 2019.
